# Supplementary material for: Demographic Factors Predict Risk of Lymph Node Involvement in Patients with Endometrial Adenocarcinoma
Source: Biology (Basel). 2023 Jul 10;12(7):982. doi: 10.3390/biology12070982 (PMC10376236; doi:10.3390/biology12070982)

Figure S1. Nomogram for predicting risk of only pelvic LN+ with tumor size in centimeters.

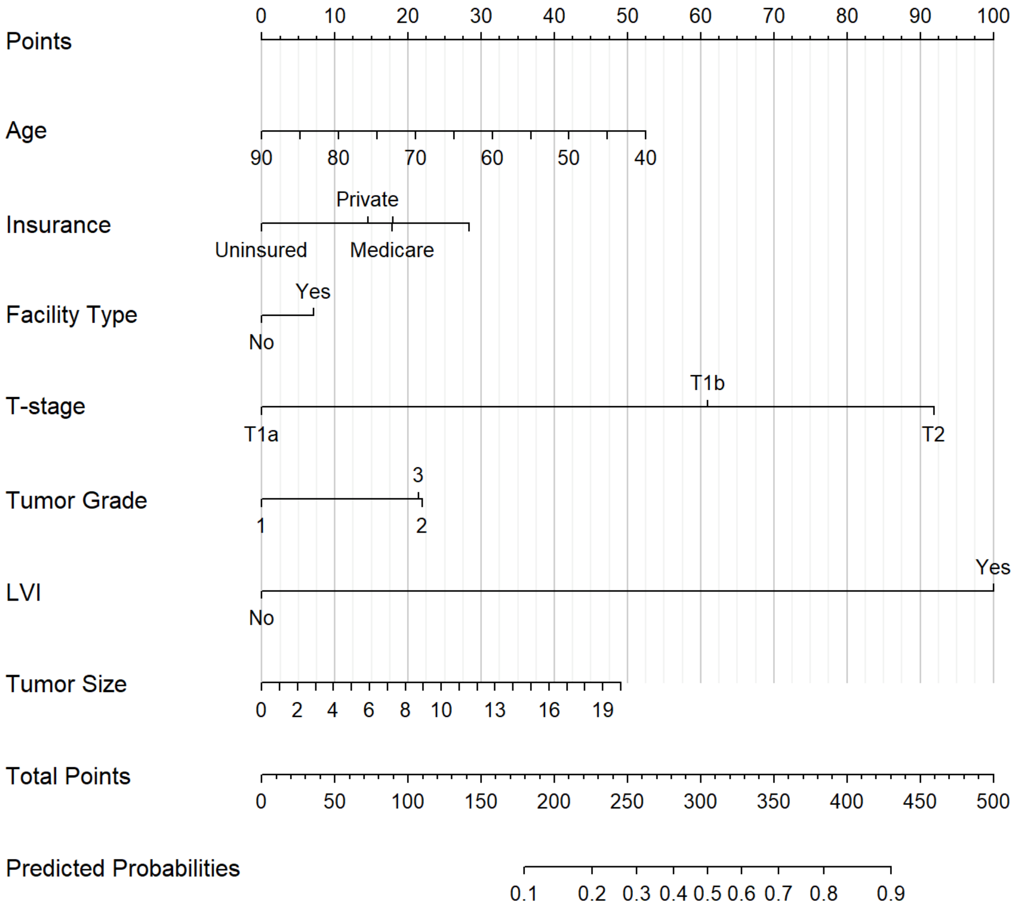

Figure S2. Nomogram for predicting risk of paraaortic +/- pelvic LN+ with tumor size in centimeters.

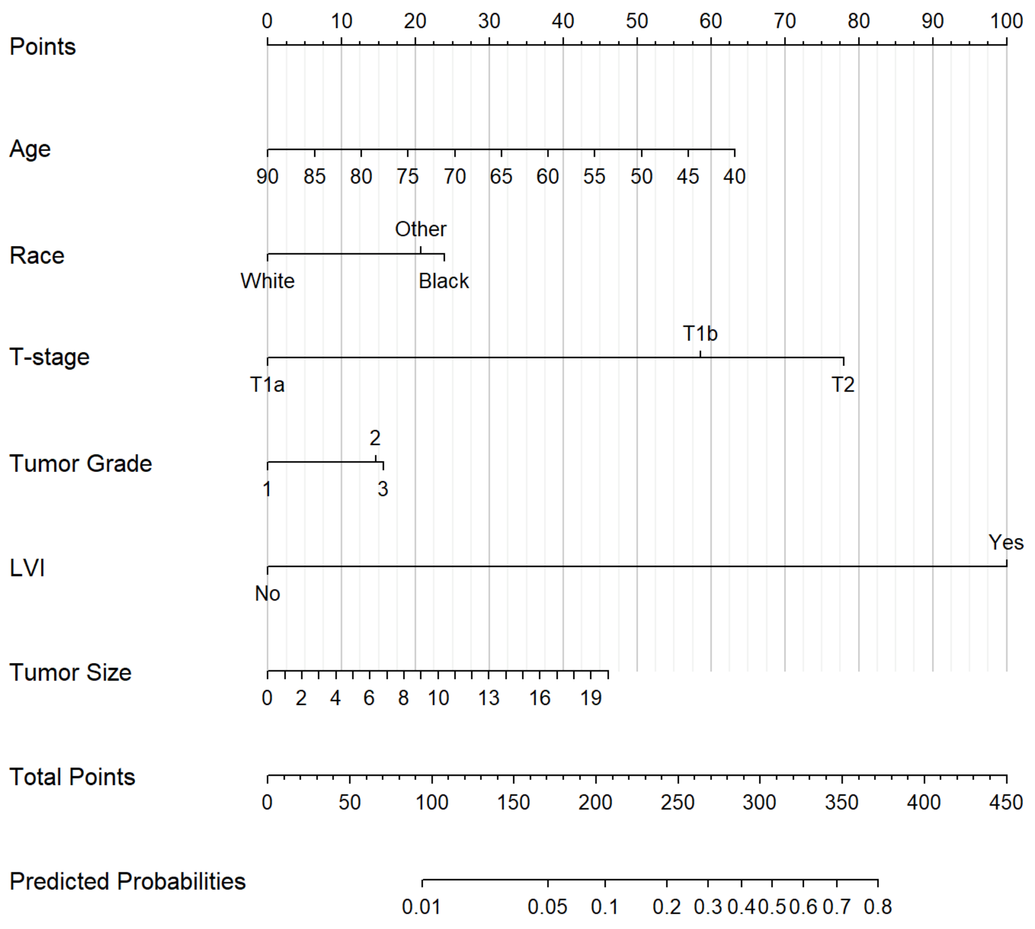

Supplement: Supplementary file 1 [file biology-12-00982-s001.zip › biology-2350707-supplementary.pdf]
